# Targeting Global Protected Area Expansion for Imperiled Biodiversity

**DOI:** 10.1371/journal.pbio.1001891

**Published:** 2014-06-24

**Authors:** Oscar Venter, Richard A. Fuller, Daniel B. Segan, Josie Carwardine, Thomas Brooks, Stuart H. M. Butchart, Moreno Di Marco, Takuya Iwamura, Liana Joseph, Damien O'Grady, Hugh P. Possingham, Carlo Rondinini, Robert J. Smith, Michelle Venter, James E. M. Watson

**Affiliations:** 1 Centre for Tropical Environmental and Sustainability Science and the School of Marine and Tropical Biology, James Cook University, Cairns, Australia; 2 School of Biological Sciences, The University of Queensland, Brisbane, Australia; 3 Global Conservation Program, Wildlife Conservation Society, New York, New York, United States of America; 4 Commonwealth Scientific and Industrial Research Organisation, Ecosystem Sciences, EcoSci Precinct, Dutton Pk, Australia; 5 International Union for Conservation of Nature, Gland, Switzerland; 6 World Agroforestry Center, University of the Philippines Los Baños, Laguna, Philippines; 7 School of Geography and Environmental Studies, University of Tasmania, Hobart, Australia; 8 BirdLife International, Cambridge, United Kingdom; 9 Global Mammal Assessment Program, Department of Biology and Biotechnologies, Sapienza Università di Roma, Rome, Italy; 10 Department of Biology and Department of Environmental Earth System Science, Stanford University, Stanford, California, United States of America; 11 Centre for Tropical Water & Aquatic Ecosystem Research, James Cook University, Cairns, Australia; 12 Department of Life Sciences, Imperial College London, Silwood Park, United Kingdom; 13 Durrell Institute of Conservation and Ecology, School of Anthropology and Conservation, University of Kent, Canterbury, United Kingdom; 14 School of Geography, Planning and Environmental Management, University of Queensland, Brisbane, Australia; Australian National University, Australia

## Abstract

Governments have agreed to expand the global protected area network from 13% to 17% of the world's land surface by 2020 (Aichi target 11) and to prevent the further loss of known threatened species (Aichi target 12). These targets are interdependent, as protected areas can stem biodiversity loss when strategically located and effectively managed. However, the global protected area estate is currently biased toward locations that are cheap to protect and away from important areas for biodiversity. Here we use data on the distribution of protected areas and threatened terrestrial birds, mammals, and amphibians to assess current and possible future coverage of these species under the convention. We discover that 17% of the 4,118 threatened vertebrates are not found in a single protected area and that fully 85% are not adequately covered (i.e., to a level consistent with their likely persistence). Using systematic conservation planning, we show that expanding protected areas to reach 17% coverage by protecting the cheapest land, even if ecoregionally representative, would increase the number of threatened vertebrates covered by only 6%. However, the nonlinear relationship between the cost of acquiring land and species coverage means that fivefold more threatened vertebrates could be adequately covered for only 1.5 times the cost of the cheapest solution, if cost efficiency and threatened vertebrates are both incorporated into protected area decision making. These results are robust to known errors in the vertebrate range maps. The Convention on Biological Diversity targets may stimulate major expansion of the global protected area estate. If this expansion is to secure a future for imperiled species, new protected areas must be sited more strategically than is presently the case.

## Abstract 2

Meeting international targets for expanding protected areas could simultaneously contribute to species conservation, but only if the distribution of threatened species informs the future establishment of protected areas.

## Author Summary

Under the Convention on Biological Diversity (CBD), governments have agreed to ambitious targets for expanding the global protected area network that could drive the greatest surge in new protected areas in history. They have also agreed to arrest the decline of known threatened species. However, existing protected areas perform poorly for coverage of threatened species, with only 15% of threatened vertebrates being adequately represented. Moreover, we find that if future protected area expansion continues in a business-as-usual fashion, threatened species coverage will increase only marginally. This is because low-cost priorities for meeting the CBD targets have little overlap with priorities for threatened species coverage. Here we propose a method for averting this outcome, by linking threatened species coverage to protected area expansion. Our analyses clearly demonstrate that considerable increases in protected area coverage of species could be achieved at minimal additional cost. Exploiting this opportunity will require directly linking the CBD targets on protected areas and threatened species, thereby formalizing the interdependence of these key commitments.

## Introduction

In 2010 the 193 parties to the Convention of Biological Diversity (CBD) adopted a new strategic plan and set of targets to tackle the continuing decline in biodiversity [1],[2]. A key element of this plan is Aichi target 11, which includes a commitment to expand the global coverage of terrestrial protected areas from the current 13% to 17% by 2020 [1]. This could drive the most rapid expansion of the global protected area network in history [3], but corresponding biodiversity benefits are far from guaranteed. This is because protected areas are often preferentially established in locations that are remote or have little agricultural value [4], failing to protect the imperiled biodiversity found on more valuable land.

Recognizing the failures of past protected area expansion, the current CBD text directs that protected areas should target places of “importance for biodiversity” that are “ecologically representative” [1]. However, these locations can be expensive to protect. For instance, the cost of expanding protected areas to cover all “important bird areas” (IBAs) has been estimated at US$58 billion annually (although these sums are still small compared to government budgets) [5]. Moreover, the majority of terrestrial regions have been identified as important for biodiversity by one or more global prioritization schemes [6], which provides myriad alternatives for meeting protected area targets in locations that are cheap. Given this, where should new protected areas be located to deliver on the Aichi biodiversity targets? One option could be based on Aichi target 12, which aims to “prevent the extinction of all known threatened species and improve and sustain their conservation status.” In situ conservation of viable populations in natural ecosystems has long been recognized as the fundamental requirement for the maintenance of biodiversity [7]. Hence measuring “biodiversity importance” in terms of protected area coverage of threatened species would help countries to simultaneously meet these two CBD targets.

Using new data from the World Database on Protected Areas [3] and distribution maps for 4,118 globally threatened birds [8], mammals [9],[10], and amphibians [10],[11], as well as ecoregions [12], we first perform a gap analysis to determine the representation of these species in the current global protected area network. We then use a systematic conservation planning framework [13] to build scenarios for cost-efficiently expanding the global protected area network to contribute to meeting the protected area and threatened species Aichi targets. Recent works have investigated strategies for achieving Aichi Target 11 by protecting IBAs [5],[14] or meeting the Global Strategy for Plant Conservation [15]. Our study is the first, to our knowledge, to use an optimization approach to develop scenarios for meeting the Aichi targets in a cost-efficient manner. Incorporating cost efficiency allows the identification of options for meeting Aichi target 11 that contribute optimally to target 12 while minimizing conflict with agricultural production.

## Methods

All spatial overlays were performed at a spatial resolution of 500 m and then aggregated into 30 km×30 km pixels to identify candidate land for protection. By processing data at the finer resolution, we are able to account for protected areas at the subpixel level, thereby minimizing omission of small-sized protected areas. This resolution of ∼⅓ degree (at the Equator) falls in the midrange between scales of ½ degree [16] and of ⅛ degree [17] typically used in such analyses.

### Protected Areas

**Figure 1:**
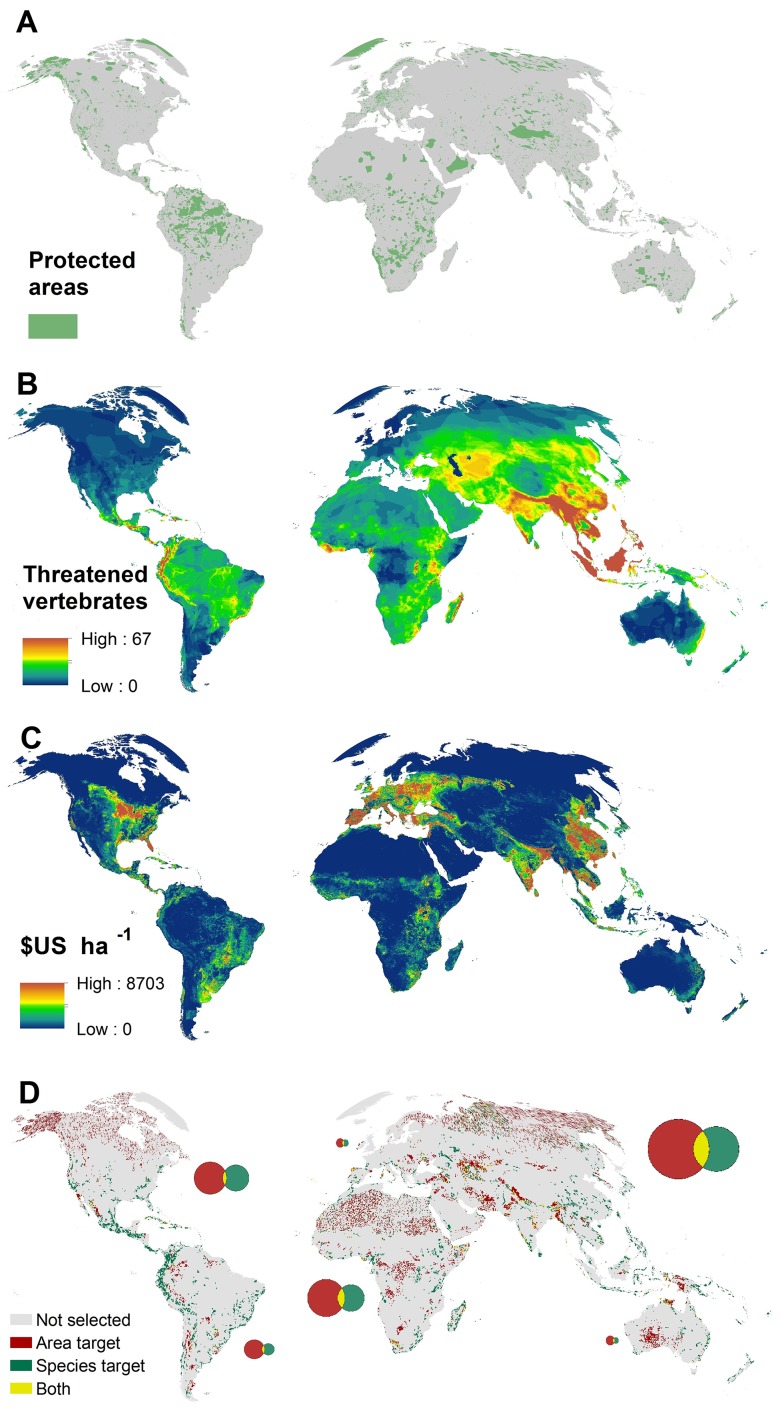
Key data inputs and output map from the systematic conservation planning framework. (A) Protected areas mapped using polygons and buffered points for nationally designated protected areas [3]. (B) The number of native and extant globally threatened terrestrial and freshwater birds [8], mammals [10], and amphibians [10] per grid square. (C) The average annual agricultural opportunity cost of protecting each 30 km grid square in 2012 $US [17]. (D) The distribution of priorities for establishing new protected areas to meet the national-level 17% targets under Aichi target 11 at minimal cost and ignoring ecological representation (red), for covering threatened species (green), and locations selected under both scenarios (yellow). The sizes of the circles in the Venn diagrams are proportional to the area required in each of the three categories.

To determine the extent of current protected areas, we extracted data on International Union for Conservation of Nature (IUCN) category I–VI protected areas from the 2012 World Database on Protected Areas [3], excluding all proposed protected areas and those lacking “national” designation. For terrestrial protected areas with a known areal extent but lacking polygonal representation, we created a circular buffer of the appropriate area around its centroid. To prevent overestimation of the areal coverage of protected areas caused by overlapping designations, we merged buffered points and polygons into a single layer. Our final protected area layer contained 135,062 protected areas covering a total of 17,026,214 km^2^, or 12.9% of the Earth's non-Antarctic land surface (Figure 1A).

### Distribution of Biodiversity

We used distribution maps for birds [8], mammals [10], and amphibians [10]. We focused on these taxa as they are the only major terrestrial taxonomic groups that have been comprehensively assessed for their distribution and extinction risk [10]. We excluded marine species and areas, noting that there are specific coverage targets for protecting the marine realm. For all three taxonomic groups, we focused on those species that are listed by the IUCN Red List as Critically Endangered, Endangered, or Vulnerable, hereafter referred to as “threatened,” resulting in 4,118 species in total (birds = 1,135, mammals = 1,107, amphibians = 1,876; Figure 1B). We focus only on threatened species as these are by definition the most likely species to go extinct, and therefore are most important for slowing biodiversity loss and contributing to CBD Aichi target 12. We excluded all portions of species ranges where the species was identified as extinct, introduced, or of uncertain origin. In addition to these data, we used data on the distribution of ecoregions as defined by the World Wildlife Fund [12].

### Protected Area Opportunity Cost

To account for the spatial variation in the cost of protected area expansion, we used a dataset on agricultural opportunity cost [18], converted to 2012 US$ and with no data values filled using regularized spline interpolation with tension (Figure 1C). The dataset provides the estimated gross agricultural rents for terrestrial areas mapped at approximately the 5 km resolution. We use these data as our surrogate for the opportunity costs of establishing new protected areas, as agricultural expansion is the greatest single cause of habitat loss, as well as the one most commonly associated with habitat loss driven by multiple factors [19],[20]. Agricultural opportunity costs also reflect the reduction in food security and tax revenue that national governments face when implementing protected areas. We applied a fixed cost of US$100 per km^2^ to reflect the transaction costs of acquiring new protected areas [21], although we recognize there is likely to be considerable spatial variation in these costs. We did not attempt to estimate the ongoing management costs of protected areas following establishment, as this metric needs to account for a number of difficult-to-measure social and socioeconomic factors [22], but a recent analysis estimated that these equate to ∼14% of the agricultural opportunity costs of protection [5].

### Gap Analysis

We assessed the occurrence of threatened vertebrates within protected areas using a representation target and an adequacy target. The representation target was achieved if any portion of the species' distribution overlapped with the protected area network. To set adequacy targets we followed the method of Rodrigues et al. [23] to scale the target to the species' overall geographic range size. Complete (i.e., 100%) coverage by protected areas was required for species with a geographic range of <1,000 km^2^. For wide-ranging species (>250,000 km^2^), the target was reduced to 10% coverage, and where geographic range size was intermediate between these extremes, the target was log-linearly interpolated.

### Scenarios for Protected Area Expansion

To explore future scenarios for the growth of the global protected area network we used the systematic conservation planning software Marxan [24]. Marxan uses a simulated annealing algorithm to select multiple alternative sets of areas that meet prespecified conservation targets (described in the following section) while trying to minimize overall cost. All spatial data on the distribution of conservation features and conservation costs were summarized into a “planning unit” layer consisting of 30 km×30 km square pixels comprising the world's non-Antarctic terrestrial areas. We intersected this planning unit layer with the protected areas and agricultural opportunity layers and the geographic distribution of each of the 4,118 threatened species and ecoregions at a 500 m resolution. This allowed us to determine the agricultural opportunity cost of the unprotected portion of each planning unit and the protected and unprotected extent of each biodiversity feature within each planning unit.

To explore the costs and benefits of alternate scenarios for achieving 17% protection of terrestrial areas, we developed four separate spatial scenarios using contrasting conservation targets. We accounted for the existing protected area network's contribution to the targets in each scenario, and then added additional protected areas to ensure all targets are met. In each scenario, the aim is to minimize the costs of meeting the conservation targets. However, to avoid the global protected area target being met only through increased protection in low-cost countries, which would reduce the total cost of the target, in all scenarios we maintain the constraint that each country must meet its national protected area target. Moreover, it is at the national level that the target is being interpreted and implemented. For each scenario, we used Marxan to perform 10 runs of 1 billion iterations each, each of which represents an alternate near optimal reserve network for meeting the relevant conservation targets at the lowest overall cost. From these 10 runs, we select and report on the results from the lowest cost solution.

#### National targets

In the first scenario, we set the conservation target as each country meeting its protected area target at the lowest agricultural opportunity cost. In this scenario, we set all countries' terrestrial protected area target to 17%, except for the 73 countries that have indicated in CBD workshops that they proposed alternative targets [25], in which case we used these targets. As countries have tended in the past to meet their targets by favoring high, far, and otherwise agriculturally low-value areas [4], we view this as our business-as-usual protected area expansion scenario. We also determine the conservation benefits of protected area target levels above the current Aichi 17% targets by setting national levels up to 30% of each country.

#### Ecoregional target

In this scenario, we maintain the national-level 17% targets from scenario a but add the additional constraint that countries meet their target in a way that ensures that each of the 821 terrestrial ecoregions receive at least 17% protection. We include this scenario as Target 11 calls for areas protected to be “ecologically representative” [1].

#### Threatened species target

In this scenario, we maintain the national-level 17% targets from scenario a but add the additional constraint that all threatened species must be covered to the level of their adequacy targets [23].

#### Threatened species preference

In this scenario, we construct an efficiency frontier between the cost of meeting the 17% target as in a and attaining threatened species conservation targets as in c. The tradeoff curve is established by iteratively increasing the value given to meeting species adequacy targets, from no value to a value equal to that given to the 17% target itself. The 17% target is always met at the national level across the tradeoff frontier.

### Commission Errors in Range Maps

The IUCN [10] and Birdlife International and NatureServe [8] range maps used in this study comprise polygons showing distribution of 4,118 globally threatened birds, mammals, and amphibians. These maps may be subject to commission errors [26]–[29], where the species is mapped as present in locations where it is in fact not present. As they affect range-based species conservation targets and lead to an overestimation of occurrence in existing or prioritized areas, commission errors could influence our study's main conclusions. We performed two analyses to determine the sensitivity of our primary results to commission errors (Text S1). First we created 100 range maps for each of the 4,118 species of birds, mammals, and amphibians that simulated commission error rates [25] by deleting 50% of the range of narrow-ranged species (range<1,000 km^2^), by deleting 25% of the range of wide-ranging species (range>250,000 km^2^), and by linearly extrapolating the deletion rate for species of intermediate ranges. Second, we identified the “Extent of Suitable Habitat” (ESH) using high-resolution species distribution models for 1,063 mammals [30]. The ESH maps were used to identify locations in the original maps for mammals that are likely to be commission errors. We then reran our analyses using (a) the maps with simulated commission errors and (b) the ESH maps, to quantify the effects of the simulated and mapped commission errors on our estimated biodiversity value of meeting the 17% protected area target, and the shape of the efficiency frontier between cost and threatened vertebrate coverage.

## Results

We find that 17% of threatened vertebrates are not found in a single protected area and 85% are not covered to the level of our adequacy targets (Figure S1A). A decade ago, 20% of globally threatened terrestrial birds, mammals, and amphibians were not found in a single protected area and 89% were inadequately protected [15]. Our analysis using updated datasets indicates that the global protected area network has made little progress since then toward securing a future for the world's threatened biodiversity.

**Figure 2:**
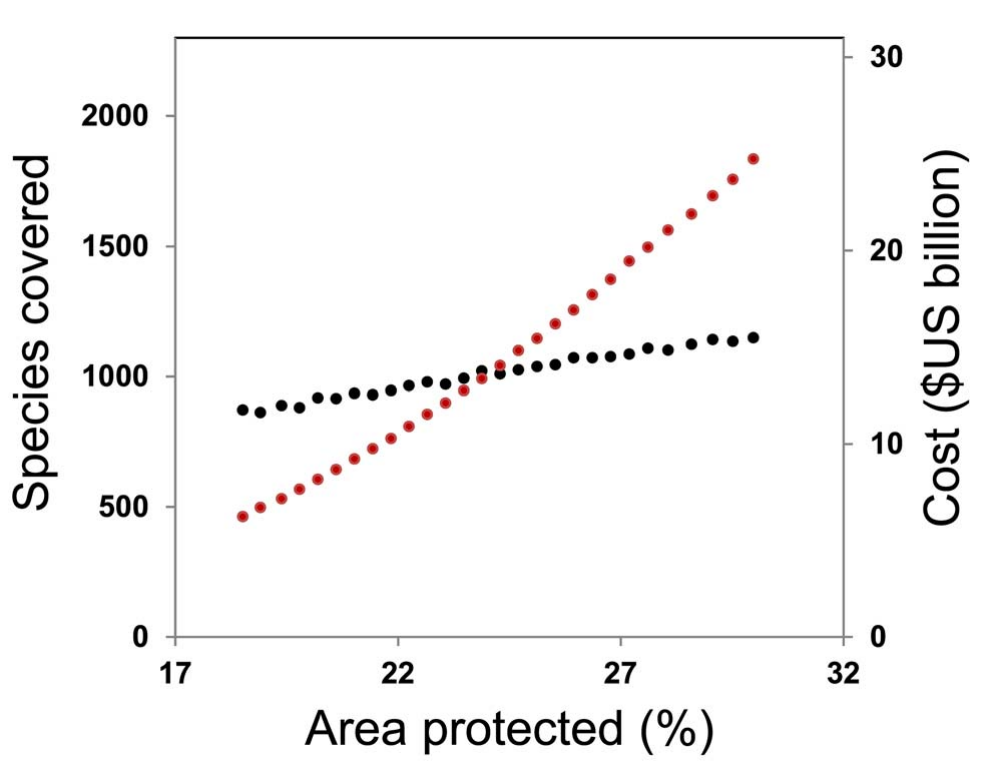
The number of globally threatened vertebrates that reach our adequacy targets (black), and the agricultural opportunity cost of establishing new protected areas (red), as the proportion of global land areas protected increases above 17%.

We discover that if countries choose to expand their protected areas in a manner that minimizes agricultural opportunity cost, meeting their national-level targets for 17% coverage would entail a once-off transaction cost of US$0.9 billion and an annual agricultural opportunity cost of $4.9 billion (Table 1). As this option aligns with the previous pattern of protected area establishment, we view it as a likely business-as-usual scenario for meeting the terrestrial coverage aspect of Aichi target 11. We find that this would result in only 852 (21%) threatened vertebrates reaching targets for adequate coverage (Figure S1B), an increase of only 249 species over existing protection (Table 1) and arguably a failure to meet Aichi target 12. Moreover, even if highly ambitious areal targets were to drive further growth of the global protected area network beyond 2020, the costs of expansion would rise steeply without providing cost-effective coverage for threatened species (Figure 2).

**Table 1:** Costs and benefits of the current protected area network and for future protection scenarios that (a) meet country-level targets for protected area coverage; (b) meet these targets while also achieving 17% protection of each terrestrial ecoregion; (c) meet the targets from scenario a and protect a scaled fraction of the geographic ranges of threatened terrestrial birds, mammals, and amphibians; and (d) achieve the country-level targets for protected area coverage while also achieving five times the level of biodiversity protection relative to scenario a.

Outcome	Current	(a) 17% Targets Nationally	(b) 17% Targets Ecoregionally	(c) Threatened Species Adequacy Target	(d) 17% Targets Nationally, with Species Preference
Area protected (km^2^ and %)*	17,026,214, 12.9%	25,816,498, 18.2%†	28,651,943, 20.2%†	28,641,412, 20.2%†	27,356,736, 19.4%†
Annual opportunity cost (+one-off transaction cost) US$ billions	na	4.92+(0.88)	24.84+(1.16)	42.54+(1.16)	7.39+(1.03)
Number (and %) of species potentially covered by protected areas	603 (15%)	852 (21%)	867 (21%)	4,118 (100%)	1,848 (45%)
Increase in species covered above current level	na	249 (41%)	264 (44%)	3,515 (580%)	1,245 (206%)

*We use all non-Antarctic land areas (132,523,065 km^2^) as our denominator when calculating proportional protection.

† Protection levels exceed 17% globally because some countries have already established protected area networks that exceed this level (Greenland, for instance, has already protected 41% of its land areas).

An alternative is to ensure a representative sample of major vegetation communities is protected, as this would protect a broader range of habitats and could lead to improved conservation outcomes. Target 11 calls for ecologically representative protected area coverage. We find that if countries meet their 17% coverage targets in a way that distributes protection across ecoregions equally, the opportunity cost of establishing the additional protected areas would be 4.5 times higher than the business-as-usual scenario ($24.8 billion annually; Table 1), but that coverage of threatened species would increase only marginally (Figure S1C). Moreover, the majority of species that reach their adequacy targets are those with a geographic range size ≥250,000 km^2^ (Figure S1C), as their wide distribution renders them more easily captured when distributing protected areas equitably across ecoregions. The species most likely to be left unprotected are narrowly distributed species, which often are those in greatest need of protection [31],[32].

These results indicate that protected area expansion targeting either the cheapest land or representation of ecoregions is not an efficient approach for covering threatened species. Alternatively, we find that locating protected areas to ensure they meet targets for adequate coverage of all 4,118 threatened species would cost about $42.5 billion annually (Table 1), which is about 7.5 times more than the cheapest option for meeting the 17% target. This difference in cost is driven by low concordance between areas that are cheap to protect and those that capture the distributions of threatened species (Figure 1D). Land selected for threatened species tends to align with tropical forest hotspots (Figure 1B), such as the tropical Andes and eastern Madagascar, whereas the cheapest land to protect is remote and often in more arid zones (Figure 1D). This lack of overlap helps explain why the existing protected area network, which has favored low-cost areas in each country [4], represents threatened species rather poorly.

**Figure 3:**
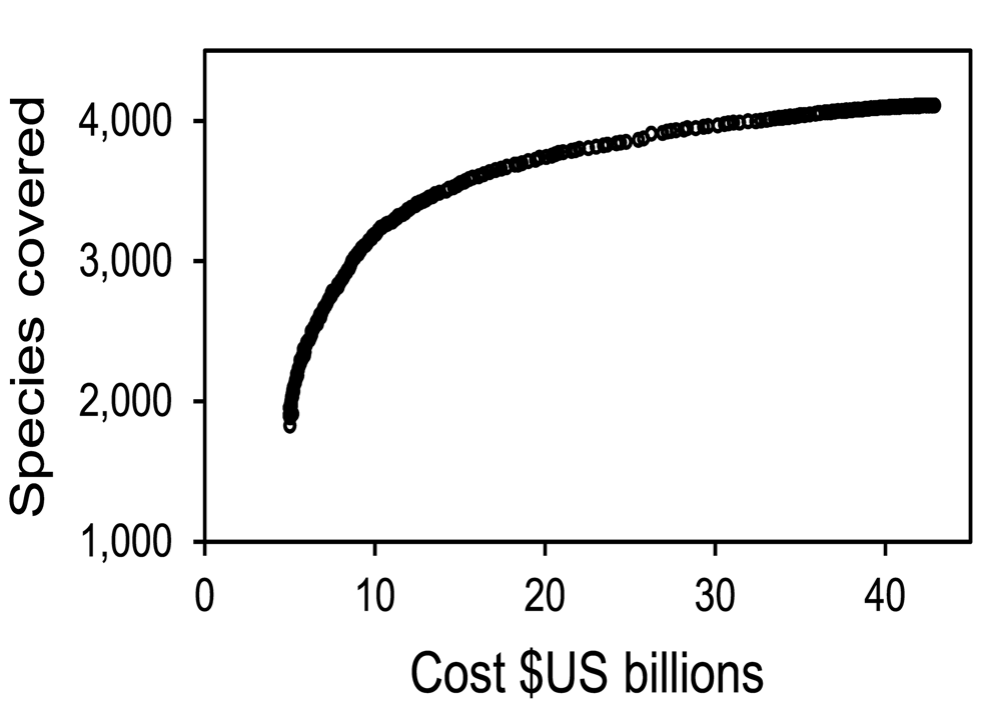
Efficiency frontier between the cost of establishing additional protected areas to achieve 17% coverage and the number of species covered. The *y*-axis presents the proportion of each species adequacy target that is met within protected areas, summed across all species, and is not directly comparable to that of the other figures, which only count species whose protected area coverage meets or exceeds their target.

How can countries reconcile the attraction of low-cost conservation with the benefits of protecting places that contribute to threatened species conservation? By varying the importance placed on meeting targets for adequate coverage of threatened species, we discover a nonlinear tradeoff between the cost of establishing additional protected areas and the proportion of threatened vertebrates covered by these areas (Figure 3). The shape of the curve illustrates that large gains in the number of species potentially protected could be achieved for relatively small increases in cost. For instance, increasing by 5-fold the number of species protected relative to the low-cost, business-us-usual scenario would increase opportunity costs to only $7.4 billion annually (1.5 times as much; Table 1).

We find that our primary results are robust to randomly simulated commission errors in the range maps. Although the number of species meeting range-based coverage targets generally decreases once commission errors are simulated (Text S1), this drop averages only 5% across the tradeoff curve (Figure S2). Moreover, both a visual interpretation and a quantitative measure of the shape of the tradeoff curve reveals that the original and commission error updated curves are similarly nonlinear. Moreover, using high-resolution expert-based habitat suitability models for 1,063 threatened mammals, we again find that commission errors are unlikely to alter our primary findings (Figure S3).

## Discussion

A small minority (15%) of threatened vertebrates are adequately covered by existing protected areas. However, the adoption of the Aichi targets marks an historic opportunity for achieving conservation of the world's biodiversity. If countries are to meet the protected area Aichi target, at least 5.8 million km^2^ of new protected areas will need to be created by 2020. Although this is a significant opportunity for biodiversity conservation, we have shown that protected area expansion that targets low-cost areas in each country and ignores threatened species is unlikely to protect such species incidentally. This remains the case even if protected areas are further expanded to cover 30% of land areas, or if they are located to cover a representative sample of Earth's terrestrial ecoregions. On the other hand, we find that if protected areas are directed in a cost-efficient manner to protect threatened vertebrates, these species could be protected for an estimated agricultural opportunity cost of about $42.5 billion annually. We also find that there is a nonlinear relationship between cost and species protection, indicating that options exist for increasing threatened species protection above the business-as-usual level at little additional cost.

Our estimate of the cost of reaching adequacy targets for all threatened birds, mammals, and amphibians is lower than the $58 billion annually estimated for protecting the world's IBAs [5], though each option comprises a similar land area. There are three primary reasons for this. First, the estimated costs of protecting IBAs include management costs, which are estimated at ∼$7 billion annually [5]. Second, IBAs are identified for their contribution to global bird conservation, without consideration of the cost of protecting these areas, whereas we used an optimization approach to identify low-cost options for meeting conservation targets [33],[34]. Third, IBAs are identified based on the presence of both threatened and nonthreatened species (e.g., congregatory species), while we focused on threatened species alone.

Our analyses are subject to a number of caveats. First, we considered relative cost based on gross agricultural rents, not management costs or the opportunity costs for other land uses [33], nor the practicalities of establishing reserves among these competing land uses. Second, overlay of coarse scale maps of species distributions onto fine-scale protected area maps generates commission errors [26],[35], though these are unlikely to qualitatively change our results. Still, as commission errors mean that species distributions overlap less than these coarse-scale maps suggest, our estimate of the area needed to protect all threatened species is a minimum [30]. Locations identified here should therefore be considered as broad indications of where specific areas for protection might be located, and our estimates of cost and the area requiring protection will be minima. Third, although we recognize that our analyses have limited taxonomic breadth, no other taxonomic groups (e.g., plants) have undergone comprehensive assessment of both extinction risk and distribution at a sufficiently fine scale for a comparable analysis [10]. Yet good indications exist from the literature that protected areas identified for broad taxonomic groups cover the majority of species in other, nontarget groups [36],[37]. Finally, our species-specific targets for protection do not account for minimum viable protected areas or connectivity and do not guarantee the long-term survival of all species. Moreover, many species are threatened by processes other than habitat loss and therefore require additional conservation actions both inside and outside protected areas [38].

For the global protected area network to fulfill its potential role as the cornerstone of biodiversity conservation [39], and for governments to meet their commitments on protected areas and species extinctions, the distribution of threatened species must inform future protected area establishment. Preventing the further loss of all threatened species is a lofty goal and will require substantial efforts. But expanding protected areas requires managing tradeoffs among societal objectives [40], and here we have shown that considerable increases in protected area coverage of species could be achieved at modest additional cost. Exploiting the nonlinearity of this tradeoff will require directly linking the Aichi targets on protected areas and threatened species (as well as other targets, including target 5 on slowing habitat loss), thereby formalizing the interdependence of these key commitments.

## Supporting Information

Figure S1 The total extant geographic range size, in logarithmic scale, and the percent of that range in protected areas for 4,118 threatened vertebrates, with the red line detailing the range-based conservation targets used in the analyses. “a” shows the protection afforded by the current protected areas, “b” shows the protection from the current network plus new protected areas necessary to meet national-level 17% targets, and “c” shows the protection from the current network plus new protected areas to meet national-level 17% targets in a way that ensures terrestrial ecoregions are protected to the level of 17%. Numbers in the graphs give the number of threatened species that have their adequacy target fully met in each scenario.
(EPS)

Figure S2 Efficiency frontier between the cost of establishing additional protected areas to achieve 17% coverage and the number of species potentially covered for the original range maps (black circles) and the randomly reduced species range maps (red stars). The *y*-axis presents the proportion of each species adequacy target that is met within protected areas, summed across all species. The red stars show the average results from 100 iterations of randomly deleting a portion of each species range; standard deviations for the 100 runs average ±0.82% across the tradeoff frontier and are therefore too small to graph.
(EPS)

Figure S3 Efficiency frontier between the cost of establishing additional protected areas to achieve 17% coverage and the number of mammal species potentially covered for original range maps (black circles) and the ESH maps (red stars). The *y*-axis presents the proportion of each species adequacy target that is met within protected areas, summed across all species.
(EPS)

Text S1 Analyses of sensitivity to range map commission errors.
(DOCX)

## Abbreviations

CBD: Convention on Biological Diversity
IUCN: International Union for Conservation of Nature
ESH: Extent of Suitable Habitat
IBAs: important bird areas

## References

[1] Convention on Biological Diversity (2011) Conference of the Parties Decision X/2: Strategic plan for biodiversity 2011–2020. www.cbd.int/decision/cop/?id=12268.

[2] Butchart SHM, Walpole M, Collen B, van Strien A, Scharlemann JPW, et al (2010) Global biodiversity: indicators of recent declines. Science 328: 1164–1168. 20430971 10.1126/science.1187512

[3] UNEP World Conservation Monitoring Centre (2012) World Database on Protected Areas. www.wdpa.org (downloaded November 2012).

[4] Joppa LN, Pfaff A (2009) High and far: biases in the location of protected areas. PLoS ONE 4 12: e8273. 20011603 10.1371/journal.pone.0008273 PMC2788247

[5] McCarthy DP, Donald PF, Scharlemann JPW, Buchanan GM, Balmford A, et al (2012) Financial costs of meeting global biodiversity conservation targets: current spending and unmet needs. Science 338: 946–949. 23065904 10.1126/science.1229803

[6] Brooks TM, Mittermeier RA, da Fonseca GAB, Gerlach J, Hoffmann M, et al (2006) Global biodiversity conservation priorities. Science 313: 58–61. 16825561 10.1126/science.1127609

[7] Convention on Biological Diversity (1992) Preamble to the Convention on Biological Diversity. http://www.cbd.int/convention/articles/default.shtml?a=cbd-00.

[8] Birdlife International and NatureServe(2012) Bird species distribution maps of the world. Version 2.0. Cambridge, UK: Birdlife International; Arlington, VA: NatureServe. www.birdlife.org (downloaded November 2012).

[9] Schipper J, Chanson JS, Chiozza F, Cox NA, Hoffmann M, et al (2008) The status of the world's land and marine mammals: diversity, threat, and knowledge. Science 322: 225–230. 18845749 10.1126/science.1165115

[10] International Union for Conservation of Nature (2012) IUCN Red List of Threatened Species. Version 2012.1. http://www.iucnredlist.org on 05/11/2012.

[11] Stuart SN, Chanson JS, Cox NA, Young BE, Rodrigues ASL, et al (2004) Status and trends of amphibian declines and extinctions worldwide. Science 306: 1783–1786. 15486254 10.1126/science.1103538

[12] Olson DM, Dinerstein E, Wikramanayake ED, Burgess ND, Powell GVN, et al (2001) Terrestrial ecoregions of the worlds: a new map of life on Earth. Bioscience 51: 933–938.

[13] Possingham HP, Ball I, Andelman S (2002) Mathematical methods for identifying representative reserve networks. In: Ferson S, Burgman MA, editors. Quantitative methods for conservation biology. New York: Springer-Verlag.

[14] Butchart SHM, Scharlemann JPW, Evans MI, Quader S, Aricò S, et al (2012) Protecting important sites for biodiversity contributes to meeting global conservation targets. PLoS ONE 7: e32529. 22457717 10.1371/journal.pone.0032529 PMC3310057

[15] Joppa L, Visconti P, Jenkins CN, Pimm SL (2013) Achieving the convention on biological diversity's goals for plant conservation. Science 341: 1100–1103. 24009391 10.1126/science.1241706

[16] Rodrigues ASL, Andelman SJ, Bakarr MI, Boitani L, Brooks TM, et al (2004) Effectiveness of the global protected area network in representing species diversity. Nature 428: 640–643. 15071592 10.1038/nature02422

[17] Strassburg BBN, Rodrigues ASL, Gusti M, Balmford A, Fritz S, et al (2012) Impacts of incentives to reduce emissions from deforestation on global species extinctions. Nature Clim Change 2: 350–355.

[18] Naidoo R, Iwamura T (2007) Global-scale mapping of economic benefits from agricultural lands: implications for conservation priorities. Biological Conservation 140: 40–49.

[19] Geist HJ, Lambin EF (2002) Proximate causes and underlying driving forces of tropical deforestation. Bioscience 52: 143–150.

[20] Achard F, Eva HD, Stibig HJ, Mayaux P, Gallego J, et al (2002) Determination of deforestation rates of the world's humid tropical forests. Science 297: 999–1002. 12169731 10.1126/science.1070656

[21] Fuller RA, McDonald-Madden E, Wilson KA, Carwardine J, Grantham HS, et al (2010) Replacing underperforming protected areas achieves better conservation outcomes. Nature 466: 365–367. 20592729 10.1038/nature09180

[22] McCreless E, Visconti P, Carwardine J, Wilcox C, Smith RJ (2013) Cheap and nasty? The potential perils of using management costs to identify global conservation priorities. PLoS ONE 8: e80893. 24260502 10.1371/journal.pone.0080893 PMC3829910

[23] Rodrigues ASL, Akcakaya HR, Andelman SJ, Bakarr MI, Boitani L, et al (2004) Global gap analysis: priority regions for expanding the global protected-area network. Bioscience 54: 1092–1100.

[24] Ball IR, Possingham HP (2000) Marxan (v 1.8.6): marine reserve design using spatially explicit anealing. User manual. Brisbane, Australia: University of Queensland.

[25] Convention on Biological Diversity (2012) Protected areas: progress in the implementation of the programme of work and achievement of Aichi biodiversity target 11 (UNEP/CBD/COP/11/26).

[26] Jetz W, Sekercioglu CH, Watson JEM (2008) Ecological correlates and conservation implications of overestimating species geographic ranges. Conservation Biology 22: 110–119. 18273953 10.1111/j.1523-1739.2007.00847.x

[27] Hurlbert AH, Jetz W (2007) Species richness, hotspots, and the scale dependence of range maps in ecology and conservation. Proc Natl Acad Sci 104: 13384–13389. 17686977 10.1073/pnas.0704469104 PMC1948922

[28] Beresford AE, Buchanan GM, Donald PF, Butchart SHM, Fishpool LDC, et al (2011) Minding the protection gap: estimates of species' range sizes and holes in the Protected Area network. Animal Conservation 14: 114–116.

[29] Hawkins BA, Rueda M, Rodrigues ASL (2008) What do range maps and surveys tell us about diversity patterns? Flolia Geobot 43: 345–355.

[30] Rondinini C, Di Marco M, Chiozza F, Santulli G, Baisero D, et al (2011) Global habitat suitability models of terrestrial mammals. Phil Trans R Soc B 366: 2633–2641. 21844042 10.1098/rstb.2011.0113 PMC3140734

[31] Cantu-Salazar L, Orme CDL, Rasmussen PC, Blackburn TM, Gaston KJ (2013) The performance of the global protected area system in capturing vertebrate geographic ranges. Biodiversity and Conservation 22: 1033–1047.

[32] Ricketts TH, Dinerstein E, Boucher T, Brooks TM, Butchart SHM, et al (2005) Pinpointing and preventing imminent extinctions. Proc Natl Acad Sci U S A 102: 18497–18501. 16344485 10.1073/pnas.0509060102 PMC1311739

[33] Balmford A, Gaston KJ, Rodrigues ASL, James A (2000) Integrating costs of conservation into international priority setting. Conservation Biology 14: 597–605.

[34] Bode M, Wilson KA, Brooks TM, Turner WR, Mittermeier RA, et al (2008) Cost-effective global conservation spending is robust to taxonomic group. Proc Natl Acad Sci U S A 105: 6498–6501. 18413614 10.1073/pnas.0710705105 PMC2359771

[35] Gaston KJ, Fuller RA (2008) Commonness, population depletion and conservation biology. Trends Ecol Evol 23: 14–19. 18037531 10.1016/j.tree.2007.11.001

[36] Moore JL, Balmford A, Brooks T, Burgess ND, Hansen LA, et al (2003) Performance of sub-Saharan vertebrates as indicator groups for identifying priority areas for conservation. Conservation Biology 17: 207–218.

[37] Su JC, Debinski DM, Jakubauskas ME, Kindscher K (2004) Beyond species richness: community similarity as a measure of cross-taxon congruence for coarse-filter conservation. Conservation Biology 18: 167–173.

[38] Ferraro PJ, Pattanayak SK (2006) Money for nothing? A call for empirical evaluation of biodiversity conservation investments. PLoS Biol 4: 482–488. 10.1371/journal.pbio.0040105 PMC1435411 16602825

[39] Margules CR, Pressey RL (2000) Systematic conservation planning. Nature 405: 243–253. 10821285 10.1038/35012251

[40] Polasky S, Nelson E, Camm J, Csuti B, Fackler P, et al (2008) Where to put things? Spatial land management to sustain biodiversity and economic returns. Biological Conservation 141: 1505–1524.

