# Supplementary material for: Targeting Global Protected Area Expansion for Imperiled Biodiversity
Source: PLoS Biol. 2014 Jun 24;12(6):e1001891. doi: 10.1371/journal.pbio.1001891 (PMC4068989; doi:10.1371/journal.pbio.1001891)
Supplement: Text S1 — Analyses of sensitivity to range map commission errors. (DOCX) [file pbio.1001891.s004.docx]

**Analyses of sensitivity to range map commission errors**

The IUCN [1] and BirdLife International and NatureServe [2] range maps used in this study comprise polygons showing distribution of 4118 globally threatened birds, mammals and amphibians. The maps are used within the IUCN Red List for estimating Extent of Occurrence (EOO). These maps do not appear to be subject to high omission errors. For amphibians, for instance, 95% of the known occurrence of 4500 amphibian species fall within or immediately adjacent to their mapped distribution [3]. However, the maps may be subject to commission errors [4,5], where the species is mapped as present in locations where it is in fact not present. As they affect range-based species conservation targets and the accuracy of estimates of occurrence in existing or prioritized areas, commission errors in the maps could influence our study’s main conclusions.

We performed two sensitivity analyses to determine the likely effects of commission errors on our estimated biodiversity value of meeting the 17% protected area target in a way that minimizes cost, and the shape of the efficiency frontier between cost and threatened vertebrate protection. The shape of the efficiency frontier between these extremes was quantified as the residuals between the efficiency frontier and the straight line joining the end points of the frontier.

1. **Simulating commission errors for vertebrates**

In our first sensitivity analysis, we performed 100 simulations of commission errors in the 4118 vertebrate range maps and quantified their influence on our studies results listed above. The simulated commission errors involved iteratively deleting a portion of all 4118 species ranges before rerunning our results with the modified ranges. Specifically, we:

1. Used the original 4118 maps to construct an efficiency frontier between the cost of meeting the 17% target (scenario ‘a’ main manuscript) and attaining threatened species conservation targets (scenario ‘c’ main manuscript). To build the efficiency frontier, we followed the same methods as the main study, which was to iteratively increased the penalty for not protecting species, then run the Marxan optimization, and repeat the process until all species are protected to the level of their range-based targets [6]. To reduce computing time, we reduced the number of scenarios constructing the trade-off curve from 500 (used for the study’s main results) to 28. This process established 28 maps of protected area networks that increasingly met the targets for the original set of vertebrates.
2. Then randomly delete a portion of each of the 4118 species’ ranges. This simulates commission errors, where each species is discovered to be absent in locations where it was mapped to be present by the original maps. Areas of species’ ranges are deleted without consideration of whether the deleted portion was within existing or proposed protected areas.

Commission errors tend vary among species, with some studies showing that larger ranging species generally have lower rates of commission errors. Jetz et al [4] found that range size was typically overestimated by about 50% for narrow ranged species and by about 25% for more widely distributed species (although the polygon maps analysed in their study are of coarser resolution to those used in this study). Similarly, Rondinini et al [7] found for a subset of mammals, occupancy of those classified as LE ‘least concern’ on the IUCN Red List’ species were overestimated by 41%, while for ‘threatened’ species occupancy was overestimated by 48%. To reflect this bias in commission errors among species, when randomizing commission errors, we deleted 50% of the protected and unprotected range of narrow ranged species (range < 1000km2) and 25% of the range wide ranging species (range > 250 000km2), and linearly extrapolated the deletion rate for species of intermediate ranges.

1. We then ran Marxan to determine the level of protection afforded to the adjusted species ranges from the 28 protected area networks identified using the maps (step 1 above). As the species had a portion of their range deleted, the range-based targets for the species were increased to account for this more restricted range [6].
2. A High Powered Computer (HPC) was used to repeat Steps 2 and 3 100 times. In total, these analyses equated to randomly creating 411,800 range maps, and performing 2,800 Marxan runs of 1 billion iterations each.
3. For each of the random sets of simulated commission errors (100 iterations of steps 2 and 3), we measure the influence of the commission errors on our study’s primary results: 1) The number of species meeting range based targets from a cost-minimizing reserve network of 17% global coverage, and 2) the shape of the efficiency frontier between minimizing cost and maximizing vertebrate coverage.

We find that our study’s main results are robust to randomly simulated commission errors in the maps. Although the number of species meeting range-based coverage targets decreases once commission errors are simulated, this drop averages only 4.73% across the 28 trade-off scenarios explored in Figure S2. By quantifying the non-linearity of the curve as the residuals between the curve and the line joining its end, we find that the original trade-off curve has an average residual value of 650.9 species above the one-to-one line, similarly, the modified curve has an average residual value of 619.8 species, indicating that these curves are similarly non-linear. Moreover, the similarity of these curves is apparent from a visual inspection of figure S2. Costs do not vary across the two curves as the reserve network is identified using range maps only.

1. **Using ‘Extent of Suitable Habitat’ to measure commission errors for mammals**

In the previous analyses, we simulated commission errors for all 4118 birds, mammals and amphibians considered in this study. This slightly reduced the number of species meeting range-based coverage targets, but it did not qualitatively change the study’s primary results. However, when reducing species’ ranges to simulate commission errors, we chose locations at random for deletion. It is likely that commission errors are actually non-random, but occur in locations holding unsuitable habitat for species or that have been degraded by human activities. For our second sensitivity analysis of commission errors, we obtained high resolution species distribution models for 1063 mammals [7]. These range maps can be considered as ‘Extent of Suitable Habitat’ [8], and are developed at the 300m resolution using species-specific information on habitat suitability and habitat degradation. By contrasting our original range maps with these ESH maps, we can discover likely locations of commission errors in the former.

To determine the effect of these commission errors on our results, we build the trade-off frontier between cost and protected area coverage of mammals using the original maps. In this case we build the efficiency frontier by iteratively increasing the penalty for not protecting mammals an intermediate 75 times. This process established 75 maps of protected area networks that increasingly met the targets for the original set of species. We then used the ESH maps to determine the coverage of the 1063 mammals.

We find that on average the ESH maps cover 53% the extent of the original maps. While these maps have considerable different extents, we find that planning using the original maps subject to commission errors does not severely limit the coverage of mammals mapped using the ESH maps. Averaged across the trade-off frontier, 94.1% of the species mapped by the ESH maps reach their range-based targets from the reserve systems identified by the EOO maps (Figure S3). It is worth noting that as the ESH maps are more restricted in total extent, and the range-based targets therefore require a higher proportional coverage of ESH. Also, the species coverage and cost do not range across the same values as the analysis using all vertebrates, as fewer species require protection when only considering mammals. The shape of the trade-off curve is visual similar when commission errors are accounted for using the ESH maps, and they are also quantitatively similar (original maps residuals averaged 71.3 species, ESH residuals averaged 64.0 species).

From these analysis we conclude that the primary results detailed in the main manuscript are likely robust to commission errors in the original maps. We have not simulated nor measured omission errors, although these are likely to occur at lower rates than commission errors [3]. We have assumed that the ESH maps give the true distribution of mammals, and are therefore able to identify locations of commission errors. However, as with every species distribution map, these maps are also likely to themselves suffer from some level of commission and omission errors [7,9].

**References:**

1. IUCN (2012) IUCN Red List of Threatened Species. Version 2012.1: Downloaded from: http://www.iucnredlist.org on 05/11/2012.

2. Birdlife International and NatureServe (2012), Bird species distribution maps of the world. Version 2.0 Birdlife International Cambridge, UK and NatureServe, Arlington, USA; www.birdlife.org (Downloaded November 2012).

3. Ficetola GF, Rondinini C, Bonardi A, Katariya V, Padoa-Schioppa E, et al. (2014) An evaluation of the robustness of global amphibian range maps. Journal of Biogeography 41: 211-221.

4. Jetz W, Sekercioglu CH, Watson JEM (2008) Ecological correlates and conservation implications of overestimating species geographic ranges. Conservation Biology 22: 110-119.

5. Hurlbert AH, Jetz W (2007) Species richness, hotspots, and the scale dependence of range maps in ecology and conservation. Proceedings of the National Academy of Sciences 104: 13384-13389.

6. Rodrigues ASL, Akcakaya HR, Andelman SJ, Bakarr MI, Boitani L, et al. (2004) Global gap analysis: Priority regions for expanding the global protected-area network. Bioscience 54: 1092-1100.

7. Rondinini C, Di Marco M, Chiozza F, Santulli G, Baisero D, et al. (2011) Global habitat suitability models of terrestrial mammals. Philosophical Transactions of the Royal Society B: Biological Sciences 366: 2633-2641.

8. Buchanan GM, Donald PF, Butchart SHM (2011) Identifying Priority Areas for Conservation: A Global Assessment for Forest-Dependent Birds. PLoS ONE 6: e29080.

9. Beresford AE, Buchanan GM, Donald PF, Butchart SHM, Fishpool LDC, et al. (2011) Minding the protection gap: estimates of species' range sizes and holes in the Protected Area network. Animal Conservation 14: 114-116.
